# Planning and problem-solving training for patients with schizophrenia: a randomized controlled trial

**DOI:** 10.1186/1471-244X-11-73

**Published:** 2011-04-28

**Authors:** Katlehn Rodewald, Mirjam Rentrop, Daniel V Holt, Daniela Roesch-Ely, Matthias Backenstraß, Joachim Funke, Matthias Weisbrod, Stefan Kaiser

**Affiliations:** 1 Section of Experimental Psychopathology, Department of General Adult Psychiatry, Centre for Psychosocial Medicine, University of Heidelberg, Germany; 2 Department of Psychology, University of Heidelberg, Germany; 3 Department of Clinical Psychology, Bürger Hospital Stuttgart, Germany; 4 Department of Psychiatry, SRH Hospital Karlsbad-Langensteinbach, Germany; 5 Psychiatric University Hospital Zurich, Switzerland

## Abstract

**Background:**

The purpose of this study was to assess whether planning and problem-solving training is more effective in improving functional capacity in patients with schizophrenia than a training program addressing basic cognitive functions.

**Methods:**

Eighty-nine patients with schizophrenia were randomly assigned either to a computer assisted training of planning and problem-solving or a training of basic cognition. Outcome variables included planning and problem-solving ability as well as functional capacity, which represents a proxy measure for functional outcome.

**Results:**

Planning and problem-solving training improved one measure of planning and problem-solving more strongly than basic cognition training, while two other measures of planning did not show a differential effect. Participants in both groups improved over time in functional capacity. There was no differential effect of the interventions on functional capacity.

**Conclusion:**

A differential effect of targeting specific cognitive functions on functional capacity could not be established. Small differences on cognitive outcome variables indicate a potential for differential effects. This will have to be addressed in further research including longer treatment programs and other settings.

**Trial registration:**

ClinicalTrials.gov NCT00507988

## Background

Cognitive deficits are important predictors of functional outcome in patients with schizophrenia [1,2]. This finding has motivated the development of different psychological treatment approaches to improve cognitive deficits, which have been subsumed under the term cognitive remediation [3]. There is now converging evidence that cognitive remediation has moderate effects on cognitive performance [4]. Importantly, these improvements can generalize to functional outcome, particularly when cognitive remediation is combined with comprehensive rehabilitation, such as vocational therapy (e.g. [5-8]).

Cognitive remediation covers a broad range of interventions that are heterogeneous with respect to a number of parameters. Importantly, there is considerable variation in the cognitive functions targeted in training programs. The dominant research focus in the 1980 s and 1990 s was on training procedures addressing a particular construct or even a specific task. Most prominently this included sustained attention based on findings in the Continuous Performance Test and executive function based on Wisconsin Card Sorting Test performance [9,10]. These studies were mostly focused on the question whether cognitive deficits can be remediated through training. Recently, more comprehensive training packages addressing a set of target functions have dominated the literature (e.g. [5,11]). This goes along with a shift in outcome measures. After many of the earlier studies sought to demonstrate improvement on the task trained, a broader effect on neuropsychological test performance has subsequently been considered a condition for improvement of patient relevant outcomes [12]. There is also a growing consensus that trials aimed at improving cognition should assess functional outcome directly or through an appropriate proxy measure [13]. Accordingly, functional outcome measures have been included in most recent trials of cognitive remediation (e.g. [14,15]).

Despite this rapidly developing body of research it is still a matter of discussion, which cognitive functions should be emphasized for successful cognitive remediation [16]. Interestingly, the earliest studies of cognitive remediation in schizophrenia have addressed this question to some extent. Wagner trained patients on a stimulus discrimination task with and without requirement for abstraction, but did not find a consistent advantage of one form of training [17]. Bellack and colleagues compared trained participants on either the Wisconsin Card Sorting Test or the Halstead Category Test. The authors could show that both groups improved on the non-trained test. However, these tasks involve strongly related cognitive operations and a differential effect on other cognitive functions was not the goal of the study [18]. Another line of research focused on strategies taught during training [19,20]. However, these latter studies have not included comparisons between training of different functions or tasks. Thus, it is still an open issue whether the training of certain specific functions is more effective than training of other functions. This question is pertinent in the clinical context, where therapists often employ a mix of training interventions adapted to setting and patients.

One strategy to approach this question is to relate change in specific cognitive functions to change in a functional outcome parameter. Recent studies have suggested that change in executive functions may best predict improvement in social or daily functioning and should thus receive emphasis in cognitive remediation [21,22]. Planning and problem-solving have received increased interest, because recent developments in the assessment of executive functions with high ecological validity have been applied to the study of patients with schizophrenia [23,24]. Interestingly, planning performance on tasks with real-world approximating interface and complexity has been associated with functional outcome and related proxy measures [25-27]. This includes overall performance on the naturalistic action test, community functioning and global assessment of functioning. These studies have suggested a particular role for planning and problem-solving in cognitive remediation, but have so far not provided direct evidence.

A more direct strategy to define target cognitive functions would employ head-to-head comparisons between training of specific cognitive functions. This approach could provide direct evidence for emphasizing specific cognitive functions over others. However, this type of comparison has only been conducted by Medalia and colleagues, who compared problem-solving training with memory training and treatment as usual in a sample of hospitalized patients with chronic schizophrenia [28]. Participants in the problem-solving remediation group worked under individual supervision with the software program *Where in the USA is Carmen Sandiego? *This educational software was selected, because it requires a range of problem-solving skills and was considered to promote intrinsic motivation. Patients who received ten sessions of problem-solving training showed greater improvement on problem-solving skills required for independent living. In contrast, patients receiving memory training did improve on the trained tasks, but not in functional outcome or executive functions [29]. Thus, this study provides direct evidence for a differential effect of different targets for intervention. However, the authors note important issues to be addressed in further research. First, it is an open question whether these findings in chronic inpatients can be generalized to less impaired patient groups. Second, final sample size was limited to less than twenty Participants in each treatment group. Third, it is an open issue whether their results pertain to the specific intervention or can be generalized to training of problem-solving in a broader sense.

Thus, the two approaches for defining the focus for cognitive remediation suggest executive functioning and more specifically planning and problem-solving as treatment targets. Planning and problem-solving can be conceived as higher executive functions, which require the integration of basic cognitive functions [30]. A crucial question is whether training of these higher-order functions strongly requiring integration provides an additional benefit over a training restricted to the basic cognitive functions (e.g. processing speed, attention, memory, lower-level executive functions). More generally, the present study addresses the question which level of cognitive functioning should be targeted.

In order to train patients on planning and problem-solving, we used the software package Plan-a-Day, which is based on an earlier concept by Funke und Krüger that has been adapted for psychiatric and neurologic patients [31]. In brief, participants are given a set of errands for one day that are described by location, time, action and importance. Participants have to interactively construct a plan for this set of errands, taking priorities and timing conflicts into account. The training can be delivered in individual and group format. In the present study, small groups of no more than five patients worked with the therapist. The comparison group trained on the basic cognitive functions processing speed, memory and attention/concentration, which have all been consistently shown to be impaired in patients with schizophrenia [32,33]. These training tasks were carefully selected to not include planning and problem-solving components.

The aim of the study was to compare the effectiveness of two different approaches to cognitive remediation: targeting planning and problem-solving versus basic cognition. To our knowledge, this type of head-to-head comparison has not been performed for cognitive remediation in a rehabilitation setting. All patients received training parallel to a three-week inpatient work therapy, which was similar for all patients. We used a measure of functional capacity as a proxy measure for functional outcome. Functional capacity is assessed under standardized conditions and has been shown to be the most consistent predictor of functional outcome [2].

The study addressed two related research hypotheses:

(1) Planning and problem-solving training leads to stronger improvement of planning ability than training of basic cognition.

(2) Planning and problem-solving training leads to stronger improvement of functional capacity than training of basic cognition.

## Methods

### Study design

We carried out a single-blind randomized trial comparing planning and problem-solving training (Plan-a-Day) with training of basic cognitive functions (processing speed, attention, memory). Participants received the training interventions in an inpatient rehabilitation setting parallel to a three-week course of inpatient work therapy. Primary outcome was functional capacity and secondary outcome performance on tests of planning and problem-solving. The trial registration number at ClinicalTrials.gov is NCT00507988.

### Participants

Participants were recruited from an inpatient rehabilitation unit at the psychiatric hospital, Karlsbad Langensteinbach, Germany. Before admission, patients were living in the community. They entered a treatment program aimed at facilitating return to work. This included patients with persistent problems after an acute illness episode as well as those with a longer illness course.

All patients entered the program as inpatients to allow intensive multimodal rehabilitation. During the initial three weeks all patients received a course of work therapy to identify strengths and weaknesses with respect to further rehabilitation and to start working on treatment targets with high priority. After this initial three week period an individual rehabilitation program was developed, which included further training and/or job searching. We chose to conduct the study during the initial three-week period, because the overall treatment program during this time period was similar for all patients and any confounding effects of other treatments in addition to the study intervention would be minimized.

Patients met the DSM-IV criteria for schizophrenia or schizoaffective disorder as confirmed by the MINI International Neuropsychiatric Interview [34]. Further inclusion criteria were (1) age between 18 and 45, (2) being in a non-acute phase of illness (defined by all PANSS positive items < 5), and (3) having an estimated IQ of 80 or above. Exclusion criteria were (1) diagnosis of a neurological disorder, (2) illicit substance use during the last month, and (3) a current comorbid Axis I disorder. Patients were enrolled in the study between August 2007 and February 2009.

### Assessment

#### Planning and problem-solving

**Figure 1:**
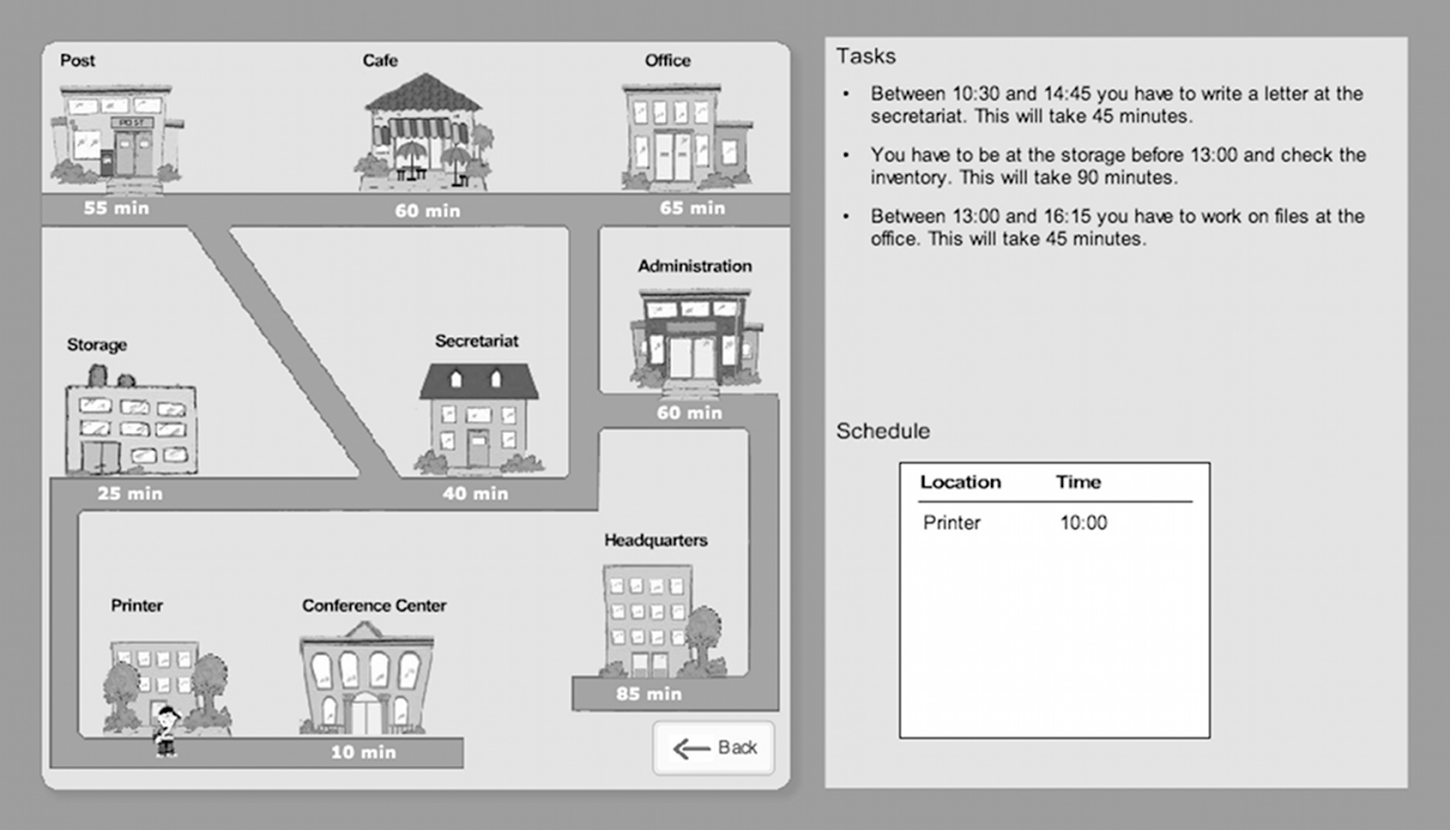
**Plan-a-Day interface**.

Planning ability was measured with a Tower of London analog (Planungstest; [35,36]) and the Zoo-Map subtest from the Behavioural Assessment of Dysexecutive Syndrome (BADS; [37,38]). Planning and problem-solving in complex scenarios was measured with a diagnostic version of Plan-a-Day [26]. This tool is a modified version of the training program (Figure 1). The diagnostic version employs a different user-interface and shorter scenarios in order to increase reliability. For diagnostic purposes, participants complete eight day plans, which take 30-45 minutes. The main scoring criterion is the total solution time. Internal consistency of the instrument has been found to be good (Cronbach's α = .78). Regarding construct validity, Plan-a-Day solution time shows significant correlations with the Tower of London and the Zoo-Map (r = 0.42, r = 0.37, both p < 0.01), but not with other neuropsychological tests. Importantly, Plan-a-Day contributes significantly to prediction of Global Assessment of Functioning scores, while other planning tests do not.

#### Functional capacity

Functional capacity was assessed with the Osnabruck Work Capabilities Profile (German: Osnabrücker Arbeitsfähigkeiten Profil, O-AFP; [39]), a 30-item inventory developed specifically for the purpose of assessing behaviour at work for persons with severe and persistent mental illness. The instrument was developed on the basis of the Work Personality Profile [40] with a stronger emphasis on easy application and sensitivity to change. The general labor market applies as a guiding principle for rating instructions. Using the O-AFP, the work therapist assessed functional capacity based on the patient's performance in work therapy at two time points, directly before the start of the intervention and directly after completion. The work therapist was trained in using the rating scale prior to the study. The O-AFP consists of three scales: "Learning Ability" (ability to use instructions and to implement changes in the work plan when necessary), "Social Communication Ability" (ability to communicate with therapists and co-workers) and "Adaptation" (ability to work reliably and to adhere to rules). Each subscale includes ten items, which are rated on a four-point rating scale. The structure of the scale was confirmed by factor analysis based on a sample of 194 patients suffering from schizophrenia or schizoaffective disorder and are shown to possess good psychometric properties [39,41]. The internal consistency (Crohnbach's α) of the three subscales is high for learning ability (α = .94), social communication ability (α = .90) and for adaptation (α = .92).The inter-rater-reliability is good (r = .81). Since the subscale "learning ability" is most closely associated with cognitive functioning, it was selected as primary outcome measure. The total score was included as a secondary outcome measure.

#### Basic cognitive functions

Working memory was assessed with the digit forward, digit backward and letter-number sequencing subtests from the WAIS III [[42]] to assess verbal memory maintenance and manipulation [42]. The Corsi Block-Tapping Task was used to assess spatial working memory maintenance and manipulation, analogous to digit span forward and backward [43]. Trail Making Test and a single-trial Stroop Test were used to assess processing speed (TMT-A and reaction time Stroop neutral condition) and inhibition (TMT-B and reaction time Stroop incongruent-neutral condition) [44,45].

Premorbid intelligence was estimated through the Mehrfachwahl-Wortschatz-Intelligenztest MWT-B, a German analog of the National Adult Reading Test [46].

### Symptoms

Symptoms were assessed by trained research psychologists using the Positive and Negative Syndrome Scale (PANSS; [47]).

### Task motivation

After completion of the intervention, we assessed task motivation for the training program with the questionnaire to assess current motivation (German: Fragebogen zur Erfassung aktueller Motivation, FAM; [48]). This questionnaire includes four subscales: interest, challenge, probability of success and anxiety.

### Interventions

#### Both groups

Participants were engaged in 10 training sessions of computer-based cognitive exercises either targeting planning and problem solving or basic cognition. The platform for all computer based exercises was the RehaCom system (Hasomed GmbH, Germany). This program system includes several adaptive therapy procedures and has been successfully used in cognitive remediation for patients with schizophrenia [49]. Following one individual introductory session, each session lasted 45 minutes and took place in a group of 3-5 participants, with participants usually completing three sessions per week for three weeks. Participants received a short introduction in every session and information about their progress after completing one session. As needed, participants received help during the training session.

#### Planning and problem-solving training

The training intervention with Plan-a-Day is based on a training concept originally developed by Kohler et al. [50]. It focuses on training participants to use a small set of simple but effective planning and decision-making heuristics (e.g. "most important tasks always first" or "maximize number or errands completed") that provide effective strategies for dealing with common goal-conflict situations in Plan-a-Day and everyday life. Increasing levels of difficulty are characterized for example by overlap between appointments, the difference between fixed and variable appointments as well as appointments, which cannot be included in the solution. In addition to computer exercises, patients included in the group working with Plan-a-Day participated in a transfer to everyday situations group. Topics in the group included, for example, work-therapy, planning shopping or planning appointments with public authorities.

#### Basic cognition training

This group trained three different tasks: (1) Processing speed: the task includes the presentation of visual stimuli that have to be responded to as quickly as possible. Increasing levels of difficulty were characterized by an increasing size of the stimulus set and progression from single to multiple choice reactions. (2) Attention and concentration: one picture shown separately has to be compared with and found among three to nine other pictures. Stimulus discriminability and set size increased with progression through levels. (3) Topological memory: the task is divided into two phases - acquisition and reproduction - of three to sixteen objects. Increasing levels of difficulty in the memory task were characterized by an increasing number of items to be retained. Patients were not instructed to use specific strategies for the basic cognition tasks.

### Procedure

The study was carried out in accordance with the Declaration of Helsinki and approved by the Ethics Committee of the University of Heidelberg Medical Faculty. All Participants gave written informed consent after the study had been fully explained. Participants were not paid for participation in the study. Following completion of the baseline assessments, participants were randomly assigned to one of the two training conditions by the project coordinator, who was not involved in the assessments or in the training procedure. Assessment of the primary outcome was blind to group allocation. All patients received work therapy parallel to the study interventions. Work therapy was conducted in a building separated from the setting of the cognitive interventions. Patients were instructed not to reveal their group allocation to the work therapist. Blinding for cognitive assessments could not be maintained in all cases.

### Statistical analysis

First, we compared the groups at baseline on the demographic, clinical, and cognitive measures using t-tests (continuous variables) and Chi-Square analyses (categorical variables). Second, in order to evaluate changes over the treatment period in cognitive functioning and functional capacity, we performed mixed analysis of variance (ANOVA) with treatment group as between subject factor and time (baseline vs. 4-week assessment) as within subject factor. In cases of non-normal distribution, the variables were log-transformed.

SPSS, Version 16, was used for statistical analyses. All statistical tests were two-tailed, and significance was determined at the alpha 0.05 level. For all analyses related to the study's specific aim, effect sizes are reported using partial eta^2^.

## Results

### Study flow

**Figure 2:**
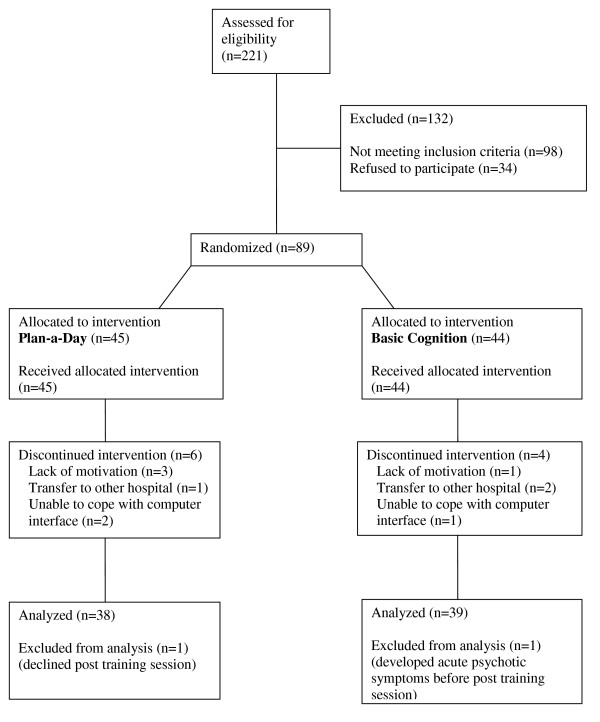
**CONSORT study flow chart**.

89 participants completed the baseline assessment and 77 (86.5%) completed the 4-week assessment. Participants completed an average of 8.42 (SD = 0.86) computer sessions. The Consort diagram is shown in Figure 2.

### Comparison of groups at baseline

Statistical tests comparing patients assigned to Plan-a-Day or Basic Cognition indicated no significant differences in any demographic, diagnostic, or baseline clinical measures. Demographic and background characteristics for each group are summarized in Table 1. All patients were treated with atypical antipsychotics. Use of anticholinergic medication did not differ significantly between Plan-a-Day and Basic Cognition groups (7.9% vs 10.3%).

**Table 1:** Demographic and clinical characteristics of patients assigned to either Plan-a-Day or Basic Cognition.

	Plan-a-Day (N = 38)		Basic Cognition (N = 39)		Test- statistic
	
Categorial Variables	N	%	N	%	Chi-Square
Gender					.65
Male	32	84.2	30	76,9	
Female	6	15.8	9	23.1	

Diagnoses					5.19
Schizophrenia, paranoid	27	71.1	30	76.9	
Schizophrenia, disorganized	1	2.6	0		
Schizophrenia, residual	2	5.3	0		
Schizophrenia, undifferentiated	1	2.6	0		
Schizoaffective disorder	5	13.2	9	23.1	
Schizophrenia simplex	2	5.3	0		

Occupational state					1.26
employed, on sick leave	13	34.2	18	46.1	
in academic or professional training, on sick leave	6	15.8	6	15.4	
unemployed	19	50	15	38.1	

**ContinuousVariables**	**Mean**	**(SD)**	**Mean**	**(SD)**	**t-statistic**

Age	28.03	7.04	29.46	7.42	-.87
Years of Education	14.68	2.96	15.55	3.71	-1.13
Premorbid IQ (MWT-B; raw score)	26.95	4.78	27.18	5.03	-.21
Age at 1^st ^hospitalization	23.03	6.28	25.70	7.05	-1.76
Global Assessment of Functioning	60.00	6.88	60.05	6.33	-.03
Baseline PANSS Total	62.03	8.72	63.79	12.57	-.72
3-week PANSS Total	54.61	7.99	56.46	9.89	-.90
QCM: challenge	20.74	3.82	19.82	3.89	1.04
QCM: interest	22.24	5.79	20.10	6.49	1.52
QCM: probability of success	14.37	2.48	14.85	2.86	-.78
QCM: anxiety	12.71	4.70	14.31	6.51	-1.24

MWT-B: Mehrfachwahl-Wortschatzintelligenz-Test Version B; PANSS: Positive and Negative Syndrome Scale; QCM: Questionnaire to assess current motivation in learning situations.

Regarding the level of functioning at the time of intake, the GAF scores indicated significant impairment (Table 1). However, in comparison with other studies of cognitive remediation, GAF scores in our sample were at the upper end of the range (e.g. [51,52]). The mean scores on cognitive test performance with available normative values (working memory tests and TMT) were within one standard deviation from the normative mean with the exception of TMT-B, which was between 1 and 2 standard deviations from the normative mean. Overall, this suggests that most of the patients included had relatively mild cognitive impairment.

### Outcomes

Outcomes are summarized in Tables 2 and 3.

**Table 2:** Primary and secondary outcome measures.

Variables	Plan-a-Day				Basic Cognition				ANOVA
	
	time: pre		post		time: pre		post		F-value interaction
	
	Mean	SD	Mean	SD	Mean	SD	Mean	SD	
**Planning and Problem-solving**									

PAD "solution time"	106.89	42.36	63.38	22.46	84.80	38.05	74.24	38.92	21.95**

Planungstest "solution time"	52.19	15.95	48.92	25.02	48.63	14.41	44.67	14.65	0.03

Zoo-Map "solution time"	111.39	58.95	97.42	52.79	105.56	59.87	99.28	42.86	0.31

**Functional capacity**									

O-AFP "learning ability"	21.16	4.87	25.42	3.74	21.59	5.36	26.08	4.16	0.07

O-AFP "total score"	68.08	9.63	80.50	8.05	69.44	10.95	80.49	8.81	0.52

Raw scores (with standard deviation) for both groups at both time points and test statistics for the interaction time (pre/post) × group.

SD: standard deviation; O-AFP: Osnabrücker Arbeitsfähigkeitenprofil (measure of functional capacity)

**: p < . 001; all other p > 0.1

**Table 3:** Basic cognition variables for both groups at both time points.

Variables	Plan-a-Day				Basic Cognition				ANOVA
	
	time: pre		post		time: pre		post		F-value interaction df = 1,75
	
	Mean	SD	Mean	SD	Mean	SD	Mean	SD	
digit span forward "Score"	9.97	1.84	10.08	1.92	9.10	2.09	9.21	2.07	<.001

digit span backward "Score"	6.71	2.03	7.47	1.90	5.72	1.72	6.36	1.72	.13

corsi forward "Score"	8.05	2.01	8.11	2.48	8.69	1.85	9.10	1.65	.66

corsi backward "Score"	7.95	1.77	7.76	2.14	7.21	1.66	7.23	2.06	.21

LNS "Score"	10.74	2.58	11.13	2.89	10.08	2.46	10.23	2.72	.32

TMT A "time"	28.74	8.97	25.51	6.99	33.03	13.23	30.83	10.52	.26

TMT B "time"	70.18	25.16	72.34	17.51	74.21	25.15	80.03	30.79	.65

Stroop neutral "time"	797.44	141.58	785.17	138.55	842.54	193.87	767.40	177.07	8.22* (df = 1,69)

Difference incongruent-neutral "time"	76.47	98.23	58.69	86.75	74.46	97.03	48.77	81.70	.14 (df = 1,69)

Exploratory analysis of the interaction time (pre/post) × group.

SD: standard deviation; LNS: Letter-Number-Sequencing; TMT:Trail Making Test

*: p < . 01; all other p > 0.1

### Planning and problem-solving

The ANOVA revealed a main effect of time for Plan-a-Day "solution time" suggesting significant improvements across both groups (F[1,75] = 71.66, p < .001, eta^2 ^= .49). Importantly, a significant time × group interaction for Plan-a-Day "solution time" was found (Table 2), indicating stronger improvement in the planning and problem solving training group. Note that this effect remains significant at a Bonferroni-corrected threshold adjusting for the five test runs for the different outcomes.

For Planungstest "solution time" we observed a significant main effect of time (F [1,75] = 7.66, p = .007, eta^2 ^= .093) indicating improvement across both groups. There was no significant effect main effect for Zoo-Map "solution time". Importantly, there were no significant time × group interactions on Planungstest and Zoo-Map (Table 2).

### Functional capacity

Analysis of change in scores for O-AFP learning ability subscale and total score did not show a significant time × group interaction, indicating a lack of significant differences between treatment groups (Table 2). A main effect of time was found for both variables (F[1,75] = 111.97, p < .001, eta^2 ^= .599 and F[1,75] = 153.26, p < .001, eta^2 ^= .671) indicating improvement in both groups during training. The numerical difference between pre- and post-training assessments was above the reliable change index cut-off for both variables (RCI learning ability = 4, RCI total score = 9).

### Exploratory analysis - basic cognition

In an exploratory analysis, each of the nine tests of basic cognition was entered into a mixed-design ANOVA (Table 3). A significant time × group interaction was found only for reaction time in the neutral condition of the Stroop task (F[1,69] = 8.22, p = .005, eta^2 ^= .11) suggesting an advantage for basic cognition training.

### Task motivation

There were no significant differences between groups on any subscale of the questionnaire used to assess training motivation (Table 1).

### Progress over the course of training

To assess the progress of participants over the course of training, we provide the mean levels reached by the group at the end of the first and last training sessions. The Plan-a-Day group progressed from level 13 (range 6-25) to level 40 (range 31-54). The basic cognition group progressed over the course of the training as follows: Memory level 5 (range 2-8) to level 10 (range 3-16), attention level 6 (range 4-8) to level 16 (range 10-20) and processing speed level 2 (range 1-3) to level 10 (4-13).

## Discussion

To our knowledge, this is the first study to compare cognitive remediation programs targeting specific cognitive functions in a rehabilitation setting. This comparison included a training of planning and problem-solving in contrast to a training of basic cognition. Overall, participants improved on cognitive performance and functional capacity. Planning and problem-solving training led to stronger improvement on one measure of planning and problem-solving, while basic cognition training had a stronger effect on one measure of processing speed. However, there was no differential effect between interventions on functional capacity. We discuss the effects observed in both training groups first and then focus on the differential effects between treatments as the main objective of the study.

Both groups improved on measures of cognitive functioning and functional capacity. We observed improvement in both patient groups in the learning ability subscale and total score of the O-AFP. The changes in O-AFP scores were above the cut-off, indicating reliable change. These findings are consistent with previous studies showing beneficial effects of programs including cognitive remediation and broader rehabilitation measures [5,7,8]. However, the interpretation of these findings is limited by the lack of a control group not receiving any cognitive intervention. Therefore, it is not clear whether our training interventions constitute a causal factor in these general improvements. The first alternative explanation to be considered is unspecific treatment effects resulting for example from hospitalization and medication. However, patients in the study were clinically stable and normally do not present short term fluctuations in performance. Another important issue is a possible effect of the intensive work therapy program on functional capacity as well as cognitive functioning. Beneficial effects of rehabilitation programs including work therapy on the OAF-P functional capacity measure have been demonstrated, although over a longer time frame [53]. Furthermore, Bell and colleagues have suggested that work therapy alone can improve cognitive functioning as it challenges memory and other cognitive functions [54]. However, to our knowledge no study has compared work therapy with a control condition in its effect on cognition.

The study's main focus was a differential effect of the training interventions on cognition and functional capacity. Regarding cognitive performance, the planning and problem-solving training lead to stronger improvement on Plan-a-Day solution time. This finding suggests that the intervention was effective at improving planning abilities. A critical objection could attribute this effect to the training of a similar task in the remediation program. However, the Plan-a-Day diagnostic and training versions differed considerably on a number of characteristics such as user interface and problem types. Therefore, although this effect might partially result from similarities between tasks, it may indicate some improvement on planning and problem-solving. There were no differential effects on the other planning tests, which address this construct on a less complex level. This difference in complexity might explain the difference in effects. In the training program, participants learn to deal with planning demands typical for real-world environments, for example involving goal conflicts requiring to skip one element. These are strategies, which are unlikely to be helpful in tasks like the Tower of London, which always have a complete and unequivocal solution.

In addition, we found a significant main effect of time for Plan-a-Day and Planungstest, suggesting that participants in both groups improved in planning ability. A critical objection would attribute this finding to a task repetition effect, although different versions of the tests were employed at both measurement points [55]. Alternatively, both the training of a more complex planning task and a set of less complex basic cognition tasks might lead to a similar improvement through different mechanisms. Overall, our results suggest that some deficits in planning and problem-solving of patients suffering from schizophrenia can be improved by a cognitive training program within three weeks. The advantage of a specific training of these functions was limited to the outcome measure most closely related to the training program. However, the improvement of the planning and problem-solving group specifically on the task most closely approaching real-world requirements suggests a potential for successful generalization to functional outcomes.

In an exploratory analysis, we addressed the issue of change in basic cognitive functions. A significant time × group interaction was only observed for reaction time in the neutral condition of the Stroop task, suggesting an advantage for basic cognition training. This result has to be viewed with caution, because we did not correct for multiple comparisons due to the exploratory character of this analysis. Reaction time in the neutral condition is a relatively pure measure of processing speed, which was also trained in the basic cognition training group. This suggests some degree of generalization across measures of processing speed, but not to other cognitive measures.

An important finding of the study is the absence of a significant differential effect of the two training programs on functional capacity. This result was observed despite the fact that the planning and problem-solving group had more contact with the trainer and explicitly practiced transfer to daily activities. Although there is meta-analytic evidence for an effect of cognitive remediation on functional outcome or respective proxy measures, this issue still remains controversial in the light of well-conducted studies with negative results [4,15]. Thus, one way to explain the absence of a differential effect would be that none of the two interventions had an effect on functional capacity.

However, Medalia et al. observed significant improvements on the Independent Living Scale specifically for the problem-solving intervention [28]. It has to be noted that our sample size was about twice as large in each treatment group and should have resulted in greater power to detect significant differences. Therefore, other differences between the studies need to be considered to explain the discrepant findings. First of all, it is important to consider similarities and differences between our intervention and the one employed by Medalia and colleagues. While both studies addressed problem-solving, our study explicitly focused on planning as a key cognitive function. In the Medalia study, planning was clearly involved in the problem-solving intervention, but a broader set of cognitive functions was likely required, although not explicitly specified. An important issue in the classification of cognitive remediation techniques is the amount of strategy teaching involved [56]. In both studies, participants in the problem-solving group were actively supported in the use of efficient problem-solving strategies. In contrast, strategies for compensating existing cognitive deficits were not explicitly trained in either study. Thus, both problem-solving interventions fill the middle ground on a continuum from drill-and-practice to compensatory approaches. Lastly, Medalia and colleagues place a strong emphasis on promoting intrinsic motivation through an engaging task environment and personal feedback. Although this was not the major theoretical background for the development of Plan-a-Day, similar elements can be found in our training task. However, in our study patients trained in small groups instead of individual training, which might have led to less individualized support and feedback. Task motivation did not differ between the two interventions, which in turn might have contributed to the observed lack of differences.

In addition, a number of factors relating to the setting and the intervention have to be considered. First, in contrast to the chronic inpatient sample in the Medalia et al. study, we included patients who were living in the community before elective admission for a treatment program promoting return to work. In addition, most patients had a relatively short duration of illness with mild impairment in cognitive functioning. A tentative interpretation of both studies would suggest that more severely impaired patients benefit more from problem-solving training in comparison to other trainings, while higher-functioning patients do not show this differential effect. Second, the duration and overall exposure to the intervention might have been too limited to produce differences between treatment groups on functional capacity. Our study was shorter than most studies of cognitive remediation (e.g. [5,6,57]), but the overall treatment exposure was larger than in the problem-solving study by Medalia et al. Nevertheless, the transfer to functional capacity in a work therapy setting might require a longer time frame. Second, in contrast to the study by Medalia, our patients participated in a broader rehabilitation program including intensive work therapy. In this enriched environment, the specific effect of a differential cognitive intervention might be more difficult to detect. Bell and colleagues have suggested that under these circumstances, a differential effect might only emerge after other treatments and supports are withdrawn [54]. Third, the control conditions differed between the two studies. In our study, the control group trained on a set of three different functions, which might have increased the effects of the basic cognition training. This combination of training targets is now implemented in most remediation programs and might be advantageous for generalization to functional outcome.

Overall, the effects of the interventions on a cognitive level were limited to measures that are relatively close but not identical to the training procedure. Whether these effects are larger and more generalized when patients receive cognitive remediation over longer time frames and in other settings remains an open issue. The lack of a differential effect on functional capacity might also result in part from the fact that both planning and processing speed have been shown to be related to functional outcome [58]. Thus, even though the interventions may affect different cognitive functions to some extent, there might be no differential effect on functional capacity. The original hypotheses that training higher levels of cognitive functioning (planning and problem-solving) provides in itself a benefit over training of basic cognition could not be confirmed.

## Conclusion

Improvements in cognitive functioning and functional capacity were observed after training of planning and problem-solving as well as basic cognition. However, no differential effect of targeting specific cognitive functions on functional capacity could be established. Small differences on cognitive outcome variables indicate a potential for differential effects. This will have to be addressed in further research including longer treatment programs and other settings. However, at present there is no conclusive evidence that training cognitive functions on different levels leads to differential improvement in patient-relevant outcome measures.

## Competing interests

JF has received royalties from Hasomed GmbH, Germany. DH and JF receive royalties for the Plan-a-Day training program from Schuhfried GmbH, Austria. All other authors declare that there are no potential conflicts of interest.

## Authors' contributions

SK, DR and MW designed the study and wrote the protocol. DH and JF developed the Plan-a-Day training and diagnostic versions. KR and MR collected the data. KR, DH and MB undertook the statistical analyses and prepared them for presentation. KR and SK wrote the first draft of the manuscript. All authors contributed to and have approved the final manuscript.

## Pre-publication history

The pre-publication history for this paper can be accessed here:

http://www.biomedcentral.com/1471-244X/11/73/prepub
